# Effects of an Intratympanic Injection of Dexamethasone Combined with Gentamicin on the Expression Level of Serum P0 Protein Antibodies in Patients with Meniere’s Disease

**DOI:** 10.6061/clinics/2020/e1622

**Published:** 2020-11-25

**Authors:** Yang Geng, Wenping Cao, Huijuan Xu, Fengfang Wu, Tao Feng

**Affiliations:** I Department of Otolaryngology, Zibo Central Hospital, Zibo, Shandong, China; II Department of Otolaryngology, Quanzhou First Hospital Affiliated to Fujian Medical University, Quanzhou, Fujian, China

**Keywords:** Dexamethasone, Gentamicin, Meniere’s Disease, Myelin P0 Protein Antibody

## Abstract

**OBJECTIVES:**

To investigate the effects of an intratympanic injection of dexamethasone combined with gentamicin on the expression level of serum P0 protein antibodies in patients with Meniere’s disease (MD).

**METHODS:**

A total of 136 patients with MD treated in our hospital were enrolled in this study. Among them, 68 patients were treated with an intratympanic injection of dexamethasone combined with gentamicin (observation group). Another 68 patients were treated with gentamicin alone (control group).

**RESULTS:**

After treatment, the expression levels of IgG and IgM in the two groups significantly decreased (*p*<0.05); the levels in the observation group were significantly lower than those in the control group (*p*<0.05). The incidences of vertigo, tinnitus, and gait instability in the observation group were significantly lower than those in the control group (*p*<0.05). Vestibular symptom index (VSI) scores in the observation group were significantly lower than those in the control group (*p*<0.05). We observed no significant difference between the two groups in the number of vertigo attacks 6 months after treatment (*p*>0.05).

**CONCLUSION:**

For patients with MD, dexamethasone combined with gentamicin can reduce the incidence of vertigo, tinnitus, and gait instability, but it has no effect on the efficacy or number of vertigo attacks 6 months after treatment. Therefore, the levels of myelin P0 protein antibodies after treatment can be used as predictors of vertigo at 6 months after treatment.

## INTRODUCTION

Meniere’s disease (MD) leads to fullness in the ear, gait problems, and nausea, with an incidence of 200-500 per 100,000 people (1,2). The quality of life of patients with MD is severely affected in the late stage. One-third of patients have bilateral MD, 74.1% of cases are accompanied by aural pressure during attacks, and 85.9% of cases are accompanied by positional vertigo. MD may be delayed by stimulation, otosclerosis, otitis media, syphilis, or trauma, which results in endolymphatic hydrops (3). MD is currently treated by surgery, drugs, and diet improvement, without precise therapeutic methods (4).

According to recent reports, the pathogenesis of MD remains unclear, but it may be related to allergy mediation, autoimmunity, circulating immune complexes, or immune heredity (5,6). Myelin, which helps action potentials to rapidly and precisely propagate along with neuronal circuits, is essential for a healthy auditory system and seriously affects hearing once damaged (7). Recent studies have shown that myelin P0 protein plays an important role in diseases of the auditory system (8,9), and it affects hearing when attacked by the autoimmune system.

Even though gentamicin as an antibiotic reduces vertigo in patients, it might also damage their vestibular function and worsen their hearing (10). Therefore, this drug is usually used for patients with MD who fail to receive conservative treatment (11). Dexamethasone is a synthetic corticosteroid with anti-inflammatory, anti-allergic, and immunosuppressive effects (12). Its significant regulatory effect on the expression of myelin P0 protein in the mouse cochlea has been shown by Maeda et al. (13). According to a study, intratympanic dexamethasone injection reduces the short-term incidence of vertigo in patients with MD, but does not reduce its long-term incidence (14). The treatment of MD with an intratympanic injection of dexamethasone combined with gentamicin has rarely been studied, and whether the combined treatment can improve the shortcomings and the therapeutic effects of the two drugs remains elusive.

Therefore, in this study, patients with MD were treated with an intratympanic injection of dexamethasone combined with gentamicin to observe its efficacy and effects on the expression levels of P0 protein antibodies, and to provide references for clinical practice.

## MATERIAL AND METHODS

### Clinical data

A total of 136 patients with MD who were diagnosed and treated in our hospital were enrolled in this study. Among them, 68 patients (39 males and 29 females with an average age of 46.6±5.4 years) were treated with an intratympanic injection of dexamethasone combined with gentamicin (observation group). Another 68 patients (33 males and 35 females with an average age of 45.4±6.1 years) were treated with gentamicin alone (control group). This study was approved by the Medical Ethics Committee of Zibo Central Hospital. All patients were informed, and they signed an informed consent form.

### Inclusion and exclusion criteria

Inclusion criteria were as follows: patients were diagnosed with MD by imaging, based on The Guideline of Diagnosis and Treatment of Meniere’s Disease issued by the French Otorhinolaryngology-Head and Neck Surgery Society in 2017 (15); patients were not allergic to therapeutic drugs; patients had complete clinical data; and patients who underwent surgery and follow-up.

Exclusion criteria were as follows: patients with congenital immune deficiency, severe infectious and inflammatory diseases, vestibular migraine, head trauma, previous ear surgery, or hepatic and renal insufficiency; and pregnant or lactating women.

### Instruments and kits

A chemiluminescence immunoassay (CLIA) analyzer (APNA370, HORIBA; Kyoto, Japan,) and matching reagents were used to detect the levels of P0 protein antibodies (IgG and IgM). Dexamethasone was purchased from Anhui Fengyuan Pharmaceutical (Hefei, China; State Food and Drug Administration (SFDA) Approval Number: H20051748) and gentamicin sulfate from Shanghai Shangyao First Biochemical Pharmaceutical (Shanghai, China; SFDA Approval Number: H31021994).

### Therapeutic methods 

The patients were placed in a supine position with their head turned to the opposite side, and a tampon with phenol glycerin (5%) was used for local anesthesia in the posterosuperior quadrant of the tympanic membrane (reference). According to the reference, Grubb, Tamara L., et al. Anesthesia and Pain Management for Veterinary Nurses and Technicians. CRC Press, 2020. For the control group, gentamicin sulfate (about 0.8 mL) was added into a 1-mL syringe connected with a No. 25 needle and pinholes were made in the anesthesia area to allow air to escape during the middle ear injection. Another pinhole was made for intratympanic injection at the same time for patients in the observation group, who received an additional intratympanic injection of dexamethasone (0.5 mL) once a day for 5 days. After the injection, the patients were placed in a supine position, with the head positioned slightly lower than the body and rotated 45° to the opposite side. The patients were advised to keep their ears upright and not swallow for 30 min after the treatment.

### Sample collection

After admission and on the sixth day after treatment, the patients’ venous blood (5 mL) was collected in pro-coagulation tubes, centrifuged at 3000 rpm for 10 min to separate the serum, and stored at -80°C until use.

### Outcome measures

Main outcome measures: The levels of IgG and IgM before and after treatment and the efficacy evaluation (17) after treatment were compared between the observation and control groups (total effective cases=markedly effective+effective cases). The efficacy evaluation criteria are shown in Table 1.

Secondary outcome measures: The clinical data, vestibular symptom index (VSI) score, dizziness handicap inventory (DHI) score, and adverse reactions after treatment were compared between the two groups. The VSI is designed to evaluate the severity of six vestibular related symptoms (dizziness, balance, nausea, vertigo, headache, and visual sensitivity) scored on a range from 0 to 10 (0 indicates no severity and 60 indicates maximum severity) Black FO, Angel CR, Pesznecker SC, Gianna C. Outcome analysis of individualized vestibular rehabilitation protocols. Am J Otololaryngol. (2000) 21:54351. The DHI comprises 25 items in three domains (functional, emotional, and physical) with three response levels. The possible total score ranges were 0-100. Higher scores indicate more severe symptoms and have been associated with increased fall risk in people with balance and vestibular disorders. Based on the number of vertigo attacks at 6 months after treatment, patients were divided into those with a high number of vertigo attacks (≥3) or a low number of vertigo attacks (<3). Receiver operating characteristic (ROC) curves were plotted to analyze the predictive values and optimal cut-off values of the levels of IgG and IgM for the vertigo attacks after treatment.

### Statistical analysis

In this study, SPSS 20.0 (IBM, Armonk, NY) was used for statistical analysis. GraphPad Prism 7 (GraphPad Software, San Diego, CA) was used to plot figures. Count data were expressed as rate (%) and analyzed by the chi-squared test. Measurement data were normally distributed and were expressed as mean±standard deviation (mean±SD). An independent-samples t-test was used for comparison between two groups, and the paired-sample t-test was used for comparison within groups. ROC curves were plotted to evaluate the predictive values of the levels of IgG and IgM for the attacks of vertigo after treatment. When *p*<0.05, the difference was statistically significant.

## RESULTS

### Comparison of clinical data

We observed no significant statistical differences between the observation and control groups in terms of sex, age, body mass index (BMI), past medical history (hypertension, diabetes, hyperlipidemia), history of smoking, history of drinking, place of residence, attacks of paroxysmal and rotatory vertigo in the past month, mean audible threshold (dB), VSI score, or DHI score (*p*>0.05) (Table 2).

### Comparison of expression levels of IgG and IgM

Before treatment, there were no significant differences between the observation and control groups in the expression levels of IgG and IgM (*p*>0.05), while after treatment, the levels in the two groups significantly decreased (*p*<0.05) (Table 3).

### Comparison of efficacy evaluation

There was no difference in the total effective rate between the observation and control groups (*p*>0.05) (Table 4).

### Comparison of adverse reactions after treatment

There were no significant differences between the observation and control groups in weakness, anorexia, and nausea and vomiting (*p*>0.05). The incidences of vertigo, tinnitus, and gait instability in the observation group were lower than those in the control group (*p*<0.05) (Table 5).

### Comparison of VSI score after treatment

**Figure 1:**
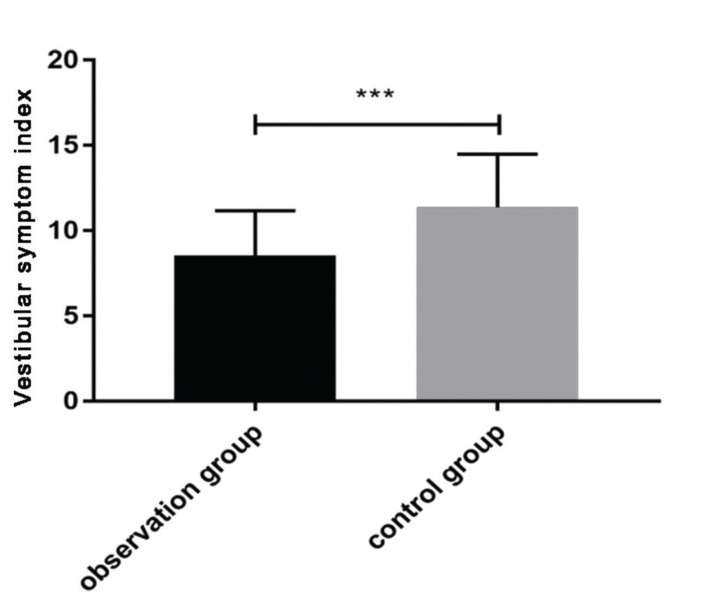
Vestibular symptom index (VSI) score after treatment. After treatment, the VSI score in the observation group was significantly lower than that in the control group (t=5.719, *p*<0.001).

After treatment, the VSI score in the observation group (8.54±2.63) was significantly lower than that in the control group (11.37±3.12) (*p*<0.05) (Figure 1).

### Comparison of DHI score after treatment

**Figure 2:**
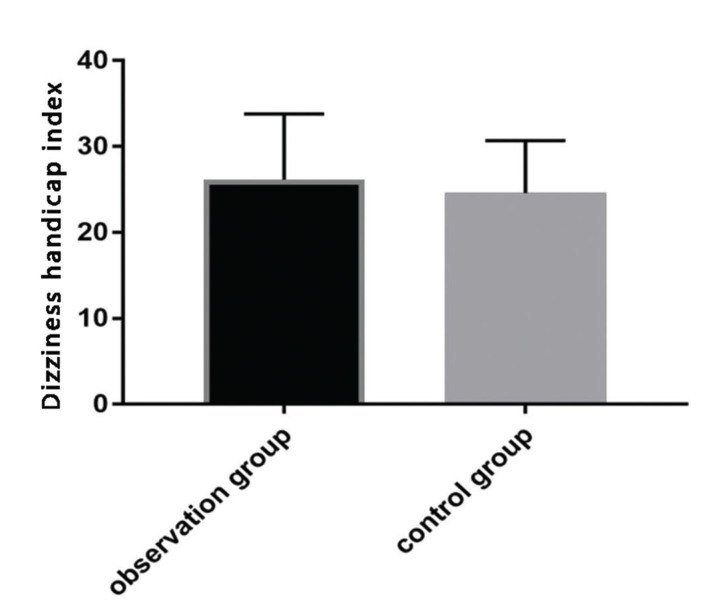
Dizziness handicap index (DHI) score after treatment. After treatment, there was no significant difference in DHI score between the observation and control groups (t=1.324, *p*=0.188).

No significant statistical difference in DHI scores between the observation group (26.14±7.63) and the control group (24.57±6.12) (*p*>0.05) was observed after the treatment (Figure 2).

### Comparing vertigo attacks 6 months after treatment

**Figure 3:**
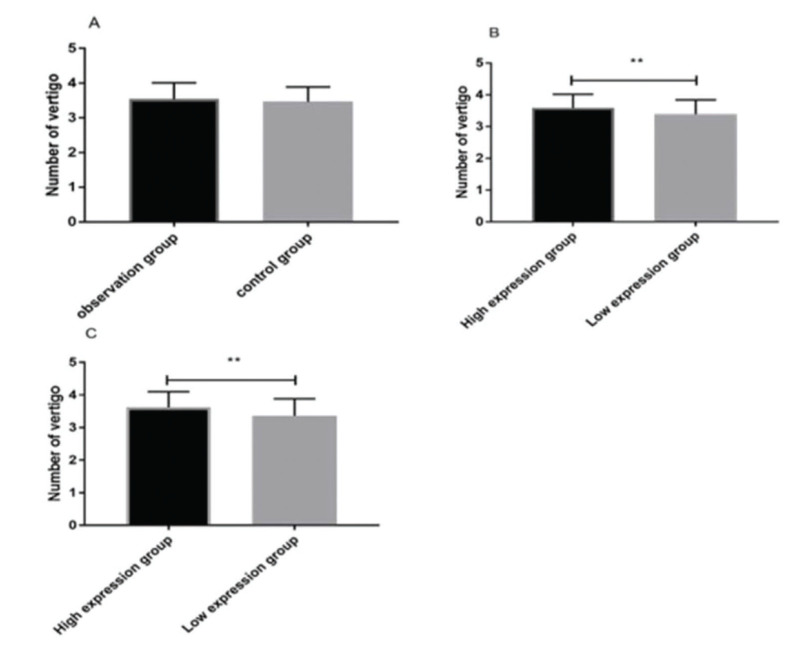
Vertigo attacks 6 months after treatment. There was no statistically significant difference in the number of vertigo attacks 6 months after treatment between the observation and control groups (t=1.036, *p*=0.302) (A). Attacks in the IgG high-expression group were significantly more frequent than those in the IgG low-expression group (t=2.650, *p*=0.009) (B). Attacks in the IgM high-expression group were significantly more frequent than those in the IgM low-expression group (t=3.030, *p*=0.003) (C). Note: ** indicates *p*<0.01.

We observed no significant statistical difference in the number of attacks of paroxysmal and rotatory vertigo 6 months after treatment between the observation group (3.54±0.47) and the control group (3.46±0.43). According to IgG and IgM expression, the patients were divided into high- and low-expression groups, respectively. Interestingly, the attacks in the IgG high-expression group (3.59±0.43) were significantly higher than those in the IgG low-expression group (3.39±0.45) (*p*<0.05). Similarly, the attacks in the IgM high-expression group (3.62±0.48) were significantly higher than those in the IgM low-expression group (3.36±0.52) (*p*<0.05) (Figure 3).

### Comparison of predictive values of IgG and IgM for vertigo attacks after treatment

**Figure 4:**
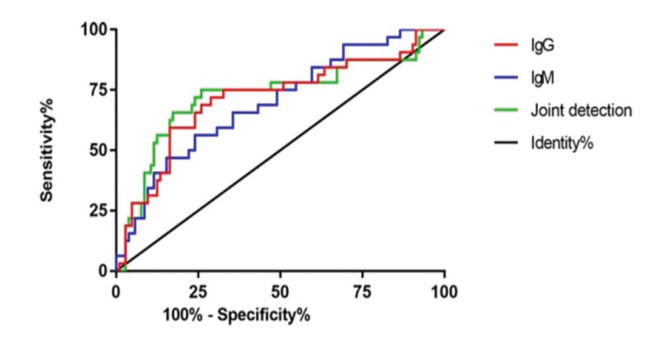
Receiver operating characteristic curves of IgG and IgM. The area under the curve (AUC) of IgG was 0.713; when the specificity was 82.69% and the sensitivity was 59.38%, the optimal cut-off value was 17.458, and the Youden index was 42.07%. The AUC of IgM was 0.696; when the specificity was 75.00% and the sensitivity was 56.25%, the optimal cut-off value was 24.020, and the Youden index was 31.25%. The AUC of the combined detection was 0.727; when the specificity was 74.04% and the sensitivity was 71.88%, the optimal cut-off value was 0.273, and the Youden index was 45.92%.

Based on vertigo attacks 6 months after treatment, patients were divided into groups according to numbers of vertigo attacks (high, ≥3, n=104; low, <3, n=32). According to the expression levels of IgG and IgM after treatment, ROC curves were plotted to analyze their predictive values for vertigo attacks. The area under the ROC curve (AUC) of IgG was 0.713 (95% confidence interval [CI] 0.602-0.824), the specificity was 82.69%, and the sensitivity was 59.38%, with a cut-off value of 17.458. The AUC of IgM was 0.696 (95% CI 0.593-0.780), the specificity was 75.00%, and the sensitivity was 56.25%, with a cut-off value of 24.020. The AUC of the combined detection was 0.727 (95% CI 0.613-0.842), the specificity was 74.04%, and the sensitivity was 71.88%, with a cut-off value of 0.273 (Figure 4 and Table 6).

## DISCUSSION

As a unique and intrinsic progressive disease, MD is difficult to diagnose due to a lack of typical manifestations, especially in its early stage (18). Its core symptoms are paroxysmal vertigo, tinnitus, and sensorineural deafness. Although its specific mechanism of pathogenesis remains unclear, recent reports suggest a plausible relationship between genetic, inflammatory, and immune factors (19-21).

In this study, the expression levels of IgG and IgM before and after treatment were compared between the observation and control groups. Before treatment, no significant differences were observed between the two groups in the expression levels of IgG and IgM, which significantly decreased after treatment, indicating a decline in the patients’ immune responses to myelin P0 protein post-treatment. Therefore, both dexamethasone and gentamicin might regulate patients’ autoimmune responses through specific pathways. Our results also showed that, after treatment, the levels of IgG and IgM in the observation group were significantly lower than those in the control group, suggesting a better response of dexamethasone combined with gentamicin than gentamicin alone in improving the immune responses to myelin P0 protein, possibly owing to the immunosuppressive effects of dexamethasone.

We then compared the therapeutic effects of gentamicin alone and in combination with dexamethasone. There was no statistically significant difference in the total effective rate between the observation group (86.76%) and the control group (83.82%). We also compared the incidence of adverse reactions after treatment, and our results showed no significant differences between the two groups in weakness, anorexia, and nausea and vomiting. The incidences of vertigo, tinnitus, and gait instability in the observation group were significantly lower than those in the control group. Therefore, the combination of dexamethasone and gentamicin can reduce the incidence of these adverse reactions after treatment. Ardıç and colleagues compared the efficacy for 25 patients with MD between dexamethasone combined with gentamicin and gentamicin alone. Their results showed that patients treated with gentamicin alone had severe high-frequency hearing loss, while patients treated with dexamethasone and gentamicin had protected auditory and vestibular cells (22), further confirming our conclusions.

In addition, VSI and DHI scores were compared after treatment, which reflect patients’ vestibular disorders and vertigo, respectively (23,24). The observation group had a significantly lower VSI score compared to the control group, while no significant difference in DHI score was observed between the two groups. At 6 months after treatment, the patients were followed up for attacks of paroxysmal and rotatory vertigo. There was no significant difference in vertigo attacks 6 months after treatment between groups. The patients were further divided depending on the expression levels of IgG and IgM after treatment into high- and low-expression groups, respectively. The number of vertigo attacks 6 months after treatment in the IgG and IgM high-expression groups were significantly higher than those in the IgG and IgM low-expression groups. Our results indicate that IgG and IgM may be predictive markers for vertigo attacks in patients with MD after treatment. According to the ROC curves, the AUC of IgG was 0.713; when the cut-off value was 17.458, the best specificity was 82.69%, and the best sensitivity was 59.38%. The AUC of IgM was 0.696; when the cut-off value was 24.020, the best specificity was 75.00%, and the best sensitivity was 56.25%. The AUC of the combined detection was 0.727; when the cut-off value was 0.273, the best specificity was 74.04%, and the best sensitivity was 71.88%. These findings further confirm that the levels of IgG and IgM after treatment can predict future vertigo attacks.

However, there were limitations to this study. First, the specific mechanisms by which dexamethasone and gentamicin regulate myelin P0 protein antibodies remain unclear and need to be fully explored. Second, corticosteroids are known to improve vestibular trauma and hearing impairment. In addition to dexamethasone, other corticosteroids such as methylprednisolone also have good therapeutic effects on MD (25), but it is not clear whether they affect the levels of myelin P0 protein antibodies, and this needs to be investigated in subsequent experiments. Lastly, vertigo attacks were counted 6 months after treatment; whether the levels of myelin P0 protein antibodies affect the attacks for a longer period is unclear and requires further analysis.

In summary, for patients with MD, dexamethasone combined with gentamicin can reduce the expression levels of myelin P0 protein antibodies and the incidences of vertigo, tinnitus, and gait instability, but with no effect on efficacy and number of vertigo attacks 6 months after treatment.

## AUTHOR CONTRIBUTIONS

Feng T designed, planned the study, collected and entered data and prepared the manuscript. Geng Y and Cao W performed data analysis and statistics. Xu H and Wu F interpreted data. Geng Y, Cao W, Xu H, and Wu F performed literature analysis and search

## Figures and Tables

**Table 1:** Efficacy evaluation criteria.

	Evaluation
Markedly effective	Improvement or deterioration of <10-dB audible threshold
Effective	>10-dB PTA improvement
Invalid	>10-dB PTA deterioration

**Table 2:** Clinical data [n (%)].

		Observation group (n=68)	Control group (n=68)	t/χ^2^	*p*
Sex				1.063	0.303
	Male	39 (57.35)	33 (48.53)		
	Female	29 (42.65)	35 (51.47)		
Age (years)		46.6±5.4	45.4±6.1	1.215	0.227
BMI (kg/m^2^)		22.64±1.82	23.01±1.97	1.138	0.257
Past medical history					
Hypertension		13 (19.12)	11 (16.18)	0.202	0.653
Diabetes		7 (10.29)	8 (11.76)	0.274	0.784
Hyperlipidemia		4 (5.88)	6 (8.82)	0.432	0.511
History of smoking				0.278	0.598
	Yes	25 (36.76)	28 (41.18)		
	No	43 (63.24)	40 (58.82)		
History of drinking				0.507	0.477
	Yes	12 (17.65)	9 (13.24)		
	No	56 (82.35)	59 (86.76)		
Place of residence				0.164	0.686
	City	53 (77.94)	51 (75.00)		
	Rural	15 (22.06)	17 (25.00)		
Attacks of paroxysmal and rotatory vertigo in the past month (frequency)		7.2±2.1	6.8±1.7	1.221	0.224
Mean audible threshold (dB)		62.71±8.21	64.52±9.43	1.194	0.235
VSI score		22.35±6.72	23.15±6.81	0.690	0.492
DHI score		62.36±13.41	58.54±11.31	1.148	0.253

BMI: body mass index, VSI: vestibular symptom index; DHI: dizziness handicap inventory.

**Table 3:** Expression levels of IgG and IgM.

	IgG (ng/L)				IgM (ng/L)			
	Before treatment	After treatment	t	*p*	Before treatment	After treatment	t	*p*
Observation group (n=68)	42.36±12.11	22.63±9.37	8.193	<0.001	36.26±14.75	25.53±9.46	5.266	<0.001
Control group (n=68)	44.32±15.06	29.32±11.79	5.528	<0.001	34.87±13.26	30.57±11.74	2.733	0.008
t	0.836	3.663			0.578	2.757		
*p*	0.404	<0.001			0.564	0.007		

**Table 4:** Efficacy evaluation [n (%)].

	Observation group (n=68)	Control group (n=68)	X^2^	*p*
Markedly effective	34 (50.00)	30 (44.12)	0.472	0.492
Effective	25 (36.76)	27 (39.71)	0.125	0.724
Invalid	9 (13.24)	11 (16.18)	0.235	0.628
Total effective	59 (86.76)	57 (83.82)		

**Table 5:** Adverse reactions after treatment [n (%)].

	Observation group (n=68)	Control group (n=68)	X^2^	*p*
Vertigo	3 (4.41)	10 (14.71)	4.168	0.041
Tinnitus	3 (4.41)	8 (11.76)	2.472	<0.001
Gait instability	2 (2.94)	10 (14.71)	5.849	0.016
Weakness	5 (7.35)	6 (8.82)	0.099	0.753
Anorexia	4 (5.88)	5 (7.35)	0.119	0.730
Nausea and vomiting	5 (7.35)	7 (10.29)	0.366	0.545

**Table 6:** Receiver operating characteristic curves.

	AUC	95%CI	Specificity	Sensitivity	Youden index	Cut-off
IgG	0.713	0.602∼0.824	82.69%	59.38%	42.07%	<7.458
IgM	0.696	0.593∼0.780	75.00%	56.25%	31.25%	<24.020
Combined detection	0.727	0.613∼0.842	74.04%	71.88%	45.92%	>0.273

AUC: area under curve, CI: confidence interval.

## References

[1] Bruderer SG, Bodmer D, Stohler NA, Jick SS, Meier CR (2017). Population-Based Study on the Epidemiology of Méniàre's Disease. Audiol Neurootol.

[2] Gürkov R, Pyykö I, Zou J, Kentala E (2016). What is Meniàre's disease? A contemporary re-evaluation of endolymphatic hydrops. J Neurol.

[3] Paparella MM (1991). Pathogenesis and pathophysiology of Meniére's disease. Acta Otolaryngol Suppl.

[4] Hussain K, Murdin L, Schilder AG (2018). Restriction of salt, caffeine and alcohol intake for the treatment of Méniàre's disease or syndrome. Cochrane Database Syst Rev.

[5] Guo SY, Zhang Y, Liu B (2018). [Recent immunology research of Meniere's disease]. Zhonghua Er Bi Yan Hou Tou Jing Wai Ke Za Zhi.

[6] Kangasniemi E, Hietikko E (2018). The theory of autoimmunity in Meniere's disease is lacking evidence. Auris Nasus Larynx.

[7] Long P, Wan G, Roberts MT, Corfas G (2018). Myelin development, plasticity, and pathology in the auditory system. Dev Neurobiol.

[8] Nadol JB, Hedley-Whyte ET, Amr SS, O Apos Malley JT, Kamakura T (2018). Histopathology of the Inner Ear in Charcot-Marie-Tooth Syndrome Caused by a Missense Variant (p.Thr65Ala) in the MPZ Gene. Audiol Neurootol.

[9] Wesdorp M, Murillo-Cuesta S, Peters T, Celaya AM, Oonk A, Schraders M (2018). MPZL2, Encoding the Epithelial Junctional Protein Myelin Protein Zero-like 2, Is Essential for Hearing in Man and Mouse. Am J Hum Genet.

[10] Patel M, Agarwal K, Arshad Q, Hariri M, Rea P, Seemungal BM (2016). Intratympanic methylprednisolone versus gentamicin in patients with unilateral Méniàre's disease: a randomised, double-blind, comparative effectiveness trial. Lancet.

[11] Naples JG, Henry L, Brant JA, Eliades SJ, Ruckenstein MJ (2019). Intratympanic Therapies in Meniere Disease: Evaluation of Outcomes and Early Vertigo Control. Laryngoscope.

[12] Crouzet E, Garcin T, Gauthier AS, He Z, Perrache C, Delavenne X (2018). Immunosuppression by a subconjunctival implant releasing dexamethasone in a rabbit model of penetrating keratoplasty. Br J Ophthalmol.

[13] Maeda Y, Fukushima K, Kariya S, Orita Y, Nishizaki K (2015). Dexamethasone Regulates Cochlear Expression of Deafness-associated Proteins Myelin Protein Zero and Heat Shock Protein 70, as Revealed by iTRAQ Proteomics. Otol Neurotol.

[14] Atrache Al Attrache N, Krstulovic C, Pérez Guillen V, Morera Pérez C, Pérez Garrigues H (2016). Response Over Time of Vertigo Spells to Intratympanic Dexamethasone Treatment in Meniere's Disease Patients. J Int Adv Otol.

[15] Nevoux J, Franco-Vidal V, Bouccara D, Parietti-Winkler C, Uziel A, Chays A (2017). Diagnostic and therapeutic strategy in Meniàre's disease. Guidelines of the French Otorhinolaryngology-Head and Neck Surgery Society (SFORL). Eur Ann Otorhinolaryngol Head Neck Dis.

[16] Ren H, Yin T, Lu Y, Kong W, Ren J (2015). Intratympanic dexamethasone injections for refractory Meniere' s disease. Int J Clin Exp Med.

[17] Masoumi E, Dabiri S, Khorsandi Ashtiani MT, Erfanian R, Sohrabpour S, Yazdani N (2017). Methylprednisolone versus Dexamethasone for Control of Vertigo in Patients with Definite Meniere's disease. Iran J Otorhinolaryngol.

[18] Zhang Y, Liu B (2017). [Advances in auditory function research of Meniere disease]. Zhonghua Er Bi Yan Hou Tou Jing Wai Ke Za Zhi.

[19] Ge NN, Shea JJ, Orchik DJ (1997). Cochlear microphonics in Méniàre's disease. Am J Otol.

[20] Paparella MM (1985). The cause (multifactorial inheritance) and pathogenesis (endolymphatic malabsorption) of Meniere's disease and its symptoms (mechanical and chemical). Acta Otolaryngol.

[21] Vambutas A, Pathak S (2016). AAO: Autoimmune and Autoinflammatory (Disease) in Otology: What is New in Immune-Mediated Hearing Loss. Laryngoscope Investig Otolaryngol.

[22] Ardıç FN, Tümkaya F, Aykal K, Çabuk B (2017). Selective Window Application of Gentamicin+ Dexamethasone in Meniere's Disease. J Int Adv Otol.

[23] Miyazaki H, Nomura Y, Mardassi A, Deveze A, Miura M, Jike M (2017). How minimally invasive vestibular neurotomy for incapacitating Meniere's disease improves dizziness and anxiety. Acta Otolaryngol.

[24] Qu YK, Huang QH, Zheng YQ, Zhong JW, Chen L, Li XH (2016). [A clinical study into the vestibular function and therapy of patients with chronic positional symptoms after acute vestibular syndrome]. Lin Chung Er Bi Yan Hou Tou Jing Wai Ke Za Zhi.

[25] Miller MW, Agrawal Y (2014). Intratympanic Therapies for Meniàre's disease. Curr Otorhinolaryngol Rep.

